# THE END OF PACE: OPPORTUNITY FOR ADVOCACY AND EDUCATION

**DOI:** 10.1093/geroni/igac059.890

**Published:** 2022-12-20

**Authors:** Gregory O'Barr, Lindsay Bonazinga, Rebecca Carey, Tracy Brosius, Anne Alexander, Barbara Dabrowski

**Affiliations:** Cheyenne Regional Medical Center, Windsor, Colorado, United States; Cheyenne Regional Medical Center, Cheyenne, Wyoming, United States; Cheyenne Regional Medical Center, Cheyenne, Wyoming, United States; Wyoming Healthworks, Cheyenne, Wyoming, United States; University of Wyoming, Laramie, Wyoming, United States; University of Wyoming, Laramie, Wyoming, United States

## Abstract

The Program for All-Inclusive Care for the Elderly (PACE) is a Medicare/Medicaid Managed Care benefit for frail older adults age 55 years or older who, although certified by the state as long-term care (LTC) eligible, choose to live independently in the community. PACE features comprehensive medical and social services coordinated by an interdisciplinary team whose goal is to promote independence. Cheyenne Regional Medical Center has served as Wyoming’s only PACE site since 2007. However, an effort to reduce Medicaid expenditures resulted in elimination of funding and closure of the program by the state. The purpose of this study was to examine the impacts of PACE closure on transition to LTC, concomitant Medicaid expenditures, as well as impacts on participant well-being. Results showed, as expected, that nursing home placement exceeded that projected, thereby increasing Medicaid expenditures in Wyoming. Additional impacts on participant well-being and advocacy benefits of GWEP partnership are discussed.

